# Effectiveness and safety of warm needle acupuncture on children with cerebral palsy: Protocol for a systematic review and meta-analysis

**DOI:** 10.1097/MD.0000000000014959

**Published:** 2019-03-15

**Authors:** Long An Chen, Hui Tuan Liu, Cihui Huang, Lu Zhang, Fangdong Zeng, Bo Xie

**Affiliations:** a Guangzhou University of Chinese Medicine; b The First Affiliated Hospital of Guangzhou University of Chinese, Guangzhou, China.

**Keywords:** children with CP, protocol, systematic review, warm needle acupuncture

## Abstract

**Background:**

Warm needle acupuncture (WNA) is an integral part of the acupuncture therapy, which combines acupuncture and moxibustion. Children with cerebral palsy (CP) is a common disease in children, which seriously affects children's growing development, physical and mental health. The clinical practice indicates that WNA has a therapeutic effect on children with CP. Here we will provide a protocol to explore the effectiveness and safety of WNA for children with CP.

**Methods:**

We will search the randomized controlled trails (RCT) literatures of WNA for children with CP in 5 English databases [PubMed, Web of Science, EMBASE, the Cochrane Central Register of Controlled Trials (Cochrane Library), and WHO International Clinical Trials Registry Platform (ICTRP)] and 4 Chinese databases [Chinese National Knowledge Infrastructure (CNKI), Chinese VIP Information, Wanfang Database, and Chinese Biomedical Literature Database (CBM)]. Activity of Daily Living Scales (ADL) of the patient will be considered as the primary outcome and the secondary outcome will include 88 items of gross motor function scale (GMFM-88), Gesell Growth Table (GGT), Criteria for judging efficacy and adverse events caused by WNA such as dizziness, nausea, vomiting, weariness, etc. The selection of the studies will be performed by EndnoteX7 software. And we will conduct all analyses with RevMan software V5.3.

**Result:**

This study will provide a rational synthesis of current evidences for WNA on children with CP.

**Conclusion:**

The conclusion of this study will provide evidence to judge the effectiveness and safety of WNA on children with CP.

**Registration:**

PROS-PERO CRD42019122034

## 1 Introduction

Cerebral palsy (CP) is a developmental disorder which results from an injury to the developing fetal or infant brain.^[1]^ Children with CP is typically made between 1year and 2 years using a combination of standardized motor assessments, neuroimaging, and a medical history.^[2]^ The pathway to children with CP is these risk factors, such as placental abnormalities, birth asphyxia, low birthweight, neonatal infections, emergency Caesarean delivery,^[3]^ maternal obesity^[4–6]^, and low socio-economic status.^[7]^ In addition, children with CP is the cause of childhood physical disability with a prevalence of 1.5 to 3.8 per 1000 live births in Europe, Australia, and the USA.^[8–10]^ According to the literature, the prevalence of CP (children with CP) is to be in the range of 1.4 to 2.3 ‰ in western civilization.^[11]^ Recent related research suggests that a significant proportion of cases have a genetic component^[12–13]^ and those with a family history of children with CP have increased risk of children with CP.^[14]^

In clinical practice, baclophenol and benzodiazepines can be used to treat children with CP. However, the use of these drugs in children with CP is challenging due to the side effects. Therefore, numerous research studies have been carried out on this topic to reduce the symptoms of children with CP with efficiency and safety.

Warm needle acupuncture (WNA) is one of the most popular traditional Chinese medicine (TCM) techniques used routinely in Asian countries, which is a combinatorial therapy of moxibustion and acupuncture. And warm acupuncture consists of acupuncture effect, warming effect and acupoint-specific stimulation.^[15]^

Through a complex mechanism of blood circulation, nervous system and immune function, WNA can achieve the goal of regulating the body and then treating diseases to be healthy.^[16]^ Some clinical research have found that WNA has a significant impact on children with CP. In addition, WNA has the advantages of safe, reliable and without toxic and side effects. However, current clinical studies show that relationships between WNA and children with CP have not been revealed clearly. Thus, the purpose of this review is to summarize clinical researches on WNA for children with CP and findings of this review will be reliable within evidence of clinical studies. This review only focuses on the effects of WNA on children with CP rather than other effective treatments.

## 2 Methods

### 2.1 Study registration

We have been registered the protocol on the International Prospective Register of Systematic Reviews (PROSPERO) (registration number, CRD42019122034) basing on the Preferred Reporting Items for Systematic Reviews and Meta-Analyses Protocols (PRISMA-P) statement guidelines on Feb 1st, 2019.

### 2.2 Inclusion criteria for study selection

#### 2.2.1 Types of studies

All available randomized controlled trials (RCTs) on WNA for children with CP will be included. Others such as retrospective study, case report, review, and studies which uses inappropriate random sequence generation methods will be excluded. Language will be restricted to Chinese and English.

#### 2.2.2 Types of participants

We will include studies on children that have been diagnosed as CP by clinicians, according to the Western medical diagnostic criteria for CP in children formulated in the Guidelines for Rehabilitation of CP in China (2015).^[17]^ There will be no restriction on age, gender, ethnicity, and profession.

#### 2.2.3 Types of interventions

The purpose of the study is on clinical trials of WNA for children with CP. Studies applied WNA in the experimental group will be included. WNA combined with other therapies will be excluded if the efficacy of WNA cannot be clarified in the combined therapy. The therapeutic intervention of controlled group can be conventional acupuncture, electro-acupuncture, auriculo-acupuncture or pharmacological therapy.

#### 2.2.4 Types of outcome measures

Primary outcome:

The Primary outcome is the Activity of Daily Living Scales (ADL).^[18]^

Secondary outcomes:

- 88 items of gross motor function scale (GMFM–88).^[19]^
- Gesell Growth Table (GGT).^[20]^
- Criteria for judging efficacy.
- Adverse events caused by WNA, such as dizziness, nausea, vomiting, weariness, etc.

### 2.3 Search methods for study identification

#### 2.3.1 Electronic searches

To identify relevant RCTs, we will search PubMed, MEDLINE, Embase, Cochrane Library, China National Knowledge Infrastructure (CNKI), Wanfang data, Chinese Biomedical Literature Database (CBM), Chinese Scientific Journals Database (VIP), and China biomedical literature database and eligible studies published up to Jan 10th, 2019 will be acquired. Various combinations of Medical Subject Headings and non-MeSH terms will be used, including “children with CP”, “CP”, “WNA”, which will be searched individually or in combination. Language, population or country restrictions will not be applied.

The specific search strategy will be (taking PubMed as an example):

1. WNA [MeSH] OR warm acupuncture [Title/Abstract] OR warm needle [Title/Abstract] OR Wen Zhen [Title/Abstract].
2. children with CP [MeSH] OR CP [Title/Abstract] OR infantile CP [Title/Abstract].
3. #1AND#2.

The strategy will be modified for other databases use if necessary. The reference lists of the relevant articles will also be checked whether they are eligible to be pooled.

#### 2.3.2 Searching other resources

Relevant systematic review or meta-analysis of RCTs will be electronically searched. Moreover, we will filter relevant medical journals and magazines to identify literature which is not included in the electronic databases.

### 2.4 Data collection and analysis

#### 2.4.1 Selection of studies

**Figure 1:**
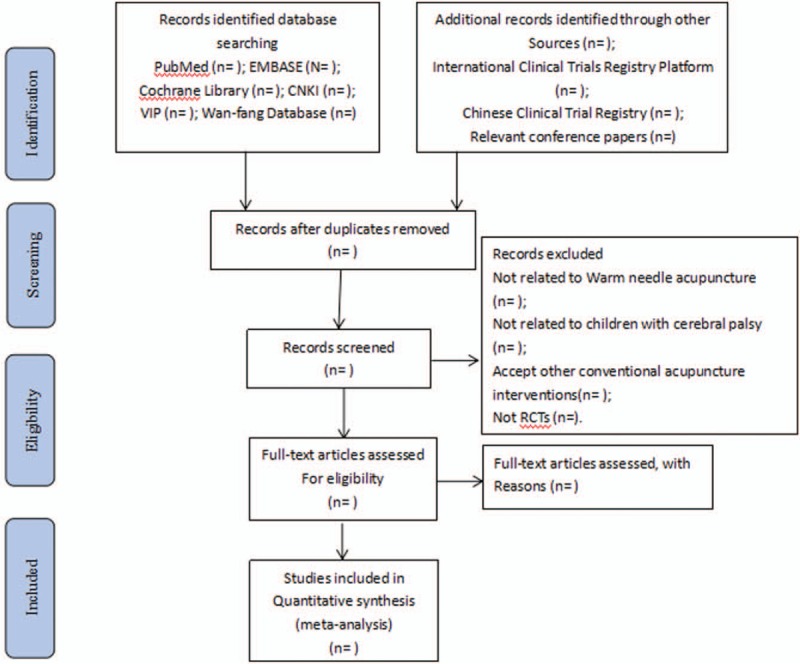
Flow diagram of study selection process. CNKI = Chinese National Knowledge Infrastructure, VIP = Chinese Science and Technology Periodicals Database.

Two researchers will import the relevant studies obtained from the databases mentioned above into EndnoteX7 that is a literature management software. And then 2 researchers will independently evaluate the titles and abstracts of the searched studies and exclude the significantly unqualified literature. Later, according to the inclusive criteria, the full text of the remaining studies will be read carefully and selected. We will resolve any different opinions generated between the 2 reviewers through discussion. When consultation fails to reach an agreement, the third reviewer will step in and provide arbitration. The study selection procedure is shown in a flow chart according to PRISMA guidelines^[21]^ (Fig. 1).

#### 2.4.2 Data extraction and management

Two independent researchers will extract the data via a standardized data collection form, after reading the full texts of each included articles. The general information such as first author, country, year of publication, design of study, basic condition of the patient, sample size and number of dropouts, duration of follow-up, details of intervention, outcome measures, and adverse events associated with WNA will be extracted and recorded. We will contact the authors to request detailed information via e-mail or telephone, if the data are ambiguous or insufficient. Any divergence on data extraction will be discussed and judged by the 2 reviewers. The third reviewer will check the final results of the data extraction, provide arbitration for further disagreements.

#### 2.4.3 Assessment of risk of bias in included studies

Two independent review authors will use the Cochrane Collaboration's risk of bias tool ^[22]^ to assess the following domains: random sequence generation, allocation concealment, blinding to participants, personnel and outcome, incomplete outcome data, selective reporting, and other biases to evaluate the risk of bias of all included studies. Through our discussion, we will resolve any discrepancies in the assessment of risk of bias and consult an arbiter if it is necessary. Finally, we will divide the quality of the studies into 3 levels: “low risk of bias”, “high risk of bias”, and “unclear risk of bias”.

#### 2.4.4 Measures of treatment effect

We will evaluate the extracted data by using mean difference (MD) for continuous variables, while using rate ratio (RR) for dichotomous variables. The confidence intervals (CI) for both continuous and dichotomous variables will be set to 95%.

#### 2.4.5 Dealing with missing data

We will try to contact the corresponding author by telephone or e-mail, for insufficient or missing trial data. In addition, we will perform a limited analysis based on the available data and discuss the potential impact of the missing data.

#### 2.4.6 Assessment of heterogeneity

Heterogeneity among the studies will be evaluated with using the *I*^2^ statistic in accordance with the Cochrane Handbook (0%–40%, might not be important, 30% to 60%, may represent moderate heterogeneity, 50% to 90%, may represent substantial heterogeneity, and 75% to 100% may represent considerable heterogeneity). We will select the random effects model, and further subgroup analysis will be performed to investigate the possible causes of heterogeneity, if the heterogeneity among trials is significant (***I***^***2***^ ≥ 50%). Conversely, we will choose the fixed effect model, if an ***I***^***2***^ values less than 50%.

#### 2.4.7 Assessment of reporting bias

We will evaluate the presence of reporting bias with funnel plots when studies are more than 10 trials. If the points of the funnel plot are dispersed and asymmetrical, we will consider that the reporting bias is existing and the reliability is low. Under the opposite conditions, the reporting bias will be considered as non-existent and the result is reliable.

#### 2.4.8 Data synthesis

We will use RevMan software V5.3 (The Nordic Cochrane Centre, The Cochrane Collaboration, Copenhagen, Denmark) to conduct all analyses. A random effects model or fixed effects model will be selected to merge the primary and secondary outcome indicators in accordance with the results of heterogeneity test. We will apply the fixed effects model for data synthesis, if the heterogeneity is low (***I***^***2***^ < 50%), while the random effects model will be conducted of the significant heterogeneity (***I***^***2***^ ≥ 50%). Differences are statistically significant if the results of Z test show that *P* value is less than .05, and for continuous variables the 95% CI does not contain 0, under the opposite conditions, for dichotomous variables the 95% CI does not contain 1.

#### 2.4.9 Subgroup analysis

With the significant heterogeneity (*I*^*2*^ ≥ 50%) and adequate trials, we will perform a subgroup analysis to explore the heterogeneity potential source, according to the difference in participant characteristics, interventions, controls, and outcome measures.

#### 2.4.10 Sensitivity analysis

We will carry out sensitivity analysis to identify the quality and robustness of the meta-analysis result when the outcome analyses involve a large degree of heterogeneity, according to sample size, methodological quality, and the effect of missing data.

#### 2.4.11 Grading the quality of evidence

We will adopt The Grading of Recommendations Assessment, Development, and Evaluation (GRADE) guidelines^[23]^ approach to assess the quality of evidence of the pooled studies. We will classify Levels of evidence quality into 4 levels: high, moderate, low, or very low.

#### 2.4.12 Ethics and dissemination

We will not need ethical approval because the data that included in our study are derived from published literature and are not linked to individual patient data. The systematic review providing implication of the effectiveness and safety of WNA for children with CP will be published in a peer-reviewed journal or conference presentations.

## 3 Discussion

CP is a permanent non-progressive cerebral lesion, which is one of the most severe brain diseases that a child can have.^[24]^ Most of children with CP are characterized by mental retardation, exacerbating their language dysfunctions and severely impacting their development and rehabilitation.^[25–26]^ Clinically, there have some side effect in the clinical treatment of children with CP. Thus, exploring effective treatment methods is the key to improving the cognitive developmental levels in children with CP.

In current research, WNA is widely applied in clinics, which has recently received significant attention in research and in practice. As is known to all, WNA is the most useful treatment which can relieve pain, improve the blood circulation and stimulate metabolism of local tissue, etc. However, the evidence of efficacy and safety of WNA is insufficient and the underlying mechanisms remain largely unknown. Thus, it is imperative to perform a systematic review and meta-analysis of available literature to evaluate the clinical efficacy and safety of WNA in children with CP.

In the current researches, the article will be the first systematic review and meta-analysis on WNA in the treatment of children with CP.

For further researches on WNA, the results of this review will provide objective statistics. In addition, the results will offer reliable references for clinicians and patients in the treatment of WNA in children with CP. More importantly, for policy makers, the results may introduce an alternative therapy of children with CP to decrease the burden and risk of public health.

## Author contributions

Bo Xie conceived the study idea. Long An Chen and Hui Tuan Liu were responsible for the design of this systematic review. CiHui Huang contributed to the data analysis plan. Lu Zhang and Fangdong Zeng drafted the manuscript and Bo Xie edited. All authors provided feedback and approved the final manuscript.

**Conceptualization:** Bo Xie.

**Data curation:** Lu Zhang.

**Methodology:** Hui Tuan Liu.

**Writing – original draft:** Long An Chen.

**Writing – review & editing:** Cihui Huang, Fangdong Zeng.
